# A Technical Note on Portal Vein Reconstruction With Interposition Cadaveric Internal Iliac Vein Graft in an Infant With Biliary Atresia and Portal Vein Hypoplasia

**DOI:** 10.7759/cureus.100585

**Published:** 2026-01-01

**Authors:** Kumudu S Solangaarachchi, Rohan Siriwardana, Suchintha B Tillakaratne, Meranthi Fernando, Aruna P Weerasuriya

**Affiliations:** 1 Hepatobiliary Surgery, Colombo North Centre for Liver Diseases, Faculty of Medicine, University of Kelaniya, Ragama, LKA; 2 Professorial Surgical Unit, North Colombo Teaching Hospital, Ragama, LKA; 3 Cardiac Surgery, Faculty of Medicine, University of Kelaniya, Ragama, LKA

**Keywords:** biliary atresia, internal iliac vein graft, kasai portoenterostomy, pediatric liver transplantation, portal vein hypoplasia, portal vein reconstruction

## Abstract

Biliary atresia (BA) represents a major indication for pediatric liver transplantation worldwide. Portal vein (PV) hypoplasia often poses technical challenges during transplantation, as it may increase the likelihood of thrombosis or anastomotic complications. We present the case of a nine-month-old infant with BA and failed Kasai portoenterostomy who underwent living donor liver transplantation. Severe PV hypoplasia necessitated interposition grafting. A 2 cm cryopreserved cadaveric internal iliac vein was used, anastomosed proximally to the superior mesenteric-splenic confluence and distally to the graft PV using 7/0 interrupted polypropylene sutures. Intraoperative Doppler confirmed portal flow of 25 cm/s with triphasic hepatic venous outflow. This case demonstrates the technical feasibility of cadaveric internal iliac vein interposition for PV reconstruction in infant BA, expanding the options for managing PV hypoplasia in pediatric liver transplantation.

## Introduction

Biliary atresia (BA) is the most common cause of neonatal cholestasis, with an incidence of one in 8,000-18,000 live births [1]. Despite a timely Kasai portoenterostomy, many children progress to end-stage liver disease requiring transplantation [1]. A significant proportion also present with portal vein (PV) hypoplasia due to progressive periportal fibrosis and diminished splanchnic flow [2].

In pediatric recipients, small vessel size increases the risk of anastomotic mismatch and thrombosis. When PV diameter is ≤3-4 mm, an interposition graft is generally required [3]. Various conduits have been explored, including autologous veins, synthetic prostheses, and cadaveric grafts [4]. While external and common iliac veins have been reported, we describe a case of PV reconstruction using a cryopreserved cadaveric internal iliac vein interposition graft in an infant with BA.

## Technical report

Case presentation

A nine-month-old girl presented with persistent jaundice from day 25 of life, pale stools, and dark urine. Laboratory investigations confirmed conjugated hyperbilirubinemia. Ultrasound failed to visualize the gallbladder. Intraoperative cholangiography during Kasai portoenterostomy (November 2024) demonstrated no passage of contrast into the intestine. Liver biopsy revealed BA with stage IV cirrhosis. The patient developed progressive jaundice, indicating Kasai failure, and was referred for transplantation. Her mother was selected as the living donor.

Surgical technique

A bilateral subcostal incision with upper midline extension was made. The falciform ligament was excised, and a Thompson retractor was applied. The liver was cirrhotic with ascites. The PV was severely hypoplastic (≤3 mm). Large coronary vein and multiple retroperitoneal collaterals were identified. Hepatic mobilization included division of the triangular and coronary ligaments, dissection of the retrohepatic inferior vena cava with ligation of small tributaries, and identification of the hepatic veins, PV, hepatic artery, and bile duct remnant. A 2 cm cryopreserved cadaveric internal iliac vein homograft was thawed according to tissue bank protocol, flushed, and inspected for defects.

Dissection of the native PV was extended proximally by mobilizing the pancreatic head to the superior mesenteric vein (SMV)-splenic vein (SV) confluence. The coronary vein shunt was ligated. The SV and SMV were individually controlled with atraumatic bulldog clamps. A generous venotomy was created at the SMV-SV confluence. The internal iliac vein graft was anastomosed to the confluence using interrupted 7/0 polypropylene sutures.

**Figure 1:**
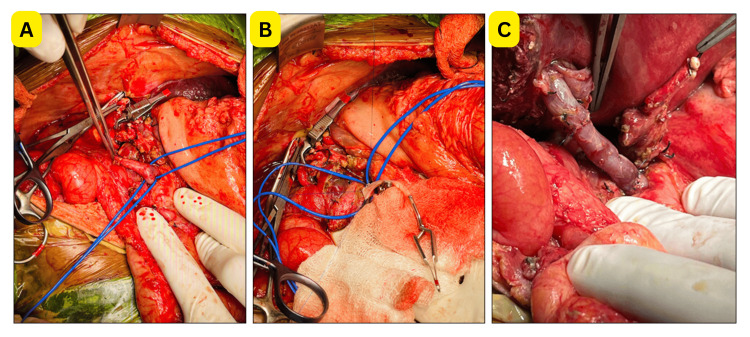
Intraoperative portal-vein reconstruction with a cadaveric interposition vein graft (A) Hypoplastic portal vein with markedly reduced caliber. (B) Cryopreserved cadaveric internal iliac vein graft, with the proximal anastomosis created at the splenic vein-superior mesenteric vein confluence to secure adequate portal inflow. (C) Completed reconstruction showing the cadaveric interposition graft establishing smooth continuity between the native portal inflow and graft portal vein

Following completion of the proximal anastomosis, the left lobe graft was implanted. The graft left hepatic vein was anastomosed to a common venotomy of the native hepatic veins with 6/0 polypropylene interrupted sutures. The PV interposition graft was then anastomosed distally to the graft PV using 7/0 interrupted polypropylene sutures, oriented to accommodate physiological graft rotation. The steps of PV reconstruction are illustrated in Figure 1.

**Figure 2:**
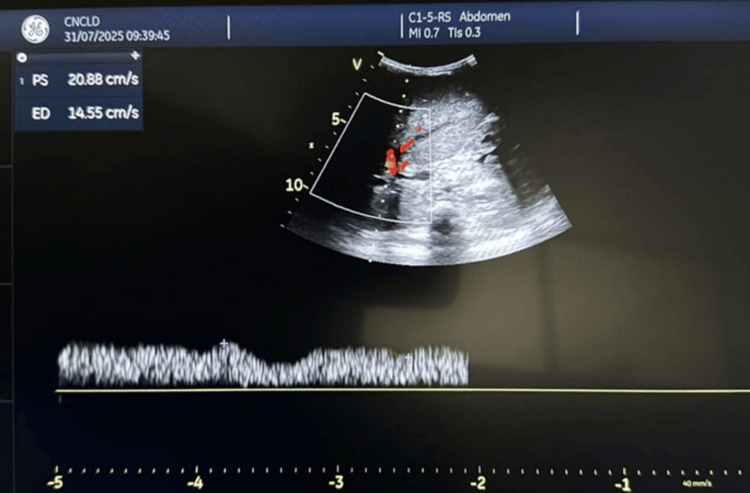
Postoperative Doppler ultrasonography confirming adequate portal-venous flow (peak velocity ≈ 25 cm/s)

Reperfusion demonstrated good graft color and turgor. Intraoperative Doppler confirmed portal flow of 25 cm/s and triphasic hepatic venous outflow. Hepatic artery reconstruction was completed with 8/0 polypropylene sutures. Postoperative Doppler ultrasonography (Figure 2) demonstrated satisfactory portal venous flow with a peak velocity of approximately 25 cm/s, confirming adequate graft perfusion.

The patient was managed in the pediatric intensive care unit (ICU) with standard anticoagulation prophylaxis and close Doppler surveillance in accordance with established pediatric liver transplantation protocols. Serial Doppler ultrasonography performed on postoperative days 1, 3, 7, and 14 consistently demonstrated adequate portal venous flow through the interposition graft, with peak velocities remaining within the expected physiological range. Immunosuppressive therapy was initiated according to institutional guidelines. Biochemical recovery was progressive and uneventful, with gradual normalisation of serum bilirubin, international normalized ratio (INR), platelet count, and liver enzyme levels, reflecting improving graft function and adequate portal perfusion. No vascular or biliary complications were observed; however, the patient developed a urinary tract infection and lung atelectasis, both of which were managed successfully. The child was subsequently discharged in stable clinical condition with a well-functioning graft and improving laboratory parameters.

## Discussion

BA accounts for the majority of pediatric liver transplants [1]. PV hypoplasia in this setting is associated with technical difficulty and high risk of early graft thrombosis [3]. In infants, direct PV anastomosis is rarely feasible when the diameter is ≤3-4 mm, making an interposition graft essential [3].

Autologous grafts (e.g., saphenous, internal jugular, inferior mesenteric, ovarian veins) are limited by small caliber, harvesting difficulty, or donor-site morbidity [5]. Synthetic conduits such as polytetrafluoroethylene (PTFE) lack compliance, do not grow with the child, and are associated with higher thrombosis and infection rates [4].

Cadaveric vein grafts offer the advantages of natural compliance, optimal size matching, and absence of donor morbidity [4]. Most published reports describe external or common iliac vein grafts for pediatric PV reconstruction [3]. In contrast, the internal iliac vein has a smaller caliber and softer texture, making it particularly suitable for infants. Cryopreservation maintains the structural integrity, and careful thawing allows safe handling for microvascular anastomosis.

In our patient, the cadaveric internal iliac vein provided a well-matched conduit, allowing tension-free, interrupted suturing with satisfactory intraoperative flow. The choice of a cryopreserved cadaveric internal iliac vein graft was guided by both existing literature and the patient’s unique anatomical constraints, particularly severe PV hypoplasia (≤3 mm). The graft was obtained from a certified tissue bank and prepared according to standard cryopreservation and thawing protocols. Short-term and sustained graft patency was confirmed by intraoperative and postoperative serial Doppler ultrasonography, and biochemical improvement correlated with adequate portal venous flow. In this case, the selection of the conduit was driven by marked PV hypoplasia unsuitable for portoplasty, the unavailability of adequate autologous options, the favorable diameter and compliance of the internal iliac vein, and its ready availability from the tissue bank. This technical report highlights important operative and postoperative considerations in the management of extreme PV hypoplasia in infants with BA.

## Conclusions

This case demonstrates that a cryopreserved cadaveric internal iliac vein can be a practical option for PV reconstruction in infants with BA and severe hypoplasia. Its small caliber, compliance, and pliability make it especially useful when autologous conduits or external/common iliac grafts are not feasible. This case report suggests fewer postoperative complications and good structural integrity compared with other graft choices, which may translate into better outcomes. Broader use and longer follow-up are still needed to confirm long-term patency and growth adaptation. This case emphasizes the importance of appropriate conduit selection, meticulous surgical technique, and structured postoperative surveillance.
